# Kinetic Characterization of Barley Straw Carbonization Using the Distributed Activation Energy Model (DAEM)

**DOI:** 10.1021/acsomega.6c01833

**Published:** 2026-07-16

**Authors:** Javier Pallarés Ranz, Cristóbal Cortés, Antonia Gil, Inmaculada Arauzo

**Affiliations:** 16765 University of Zaragoza - ENERGAIA Research Institute, Mariano Esquillor Gómez 15, Zaragoza 50018, Spain

## Abstract

As a part of modeling the production process of activated biocarbon from barley straw, a model of the initial pyrolysis or carbonization stage was developed. Material samples were divided into five particle size ranges and pyrolyzed in a thermobalance at 900 °C under a nitrogen atmosphere with five different constant heating rates. For the modeling, after a brief review of the possible strategies, the Distributed Activation Energy Model (DAEM) was chosen as the most adequate. In particular, the distribution-free variant proposed by Miura and Maki was applied, allowing the activation energy distribution and the pre-exponential factor to be directly derived from experimental data without assuming a predefined analytical form for the energy distribution. The model implementation incorporates a new approximation for the temperature integral and uses five heating rates instead of the customary three. All this results in greater accuracy and detailed characterization, previously unavailable, of a widespread biomass material. A full description of the model hypotheses and procedures is given, and the results are discussed in detail, showing good agreement between experimental data and model predictions. Perspectives for the generalized use of the model, e.g., in CFD contexts, are also discussed, highlighting its robustness and computational efficiency for engineering applications.

## Graphical abstract

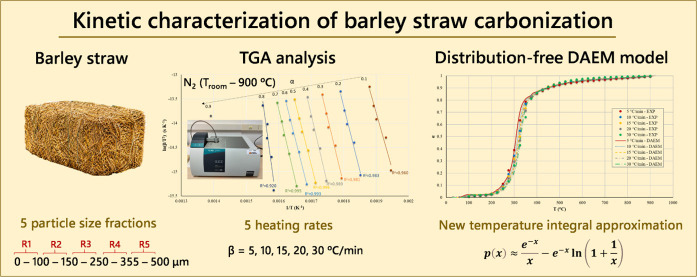

## 1 Introduction

The valorization of residual biomass through its conversion into biosustainable functional materials plays a key role in the transition to a circular economy, as it contributes to reducing dependence on fossil fuels, mitigating climate change’s effects, and promoting resource reuse and waste minimization. Activated biocarbon’s high specific surface area and adsorption capacity make it highly suitable for water and gas cleaning and purification systems. Furthermore, its importance in the energy sector has increased in recent years, with its use in CO_2_ capture technologies, hydrogen storage systems, and the development of lithium-ion batteries and supercapacitors, positioning it as a strategic material within the energy transition.

Activated carbon from fossil precursors, such as coal or coke, involves significant environmental impacts. In this context, residual lignocellulosic biomass is especially promising due to its abundance, low cost, and renewability. This work focuses on barley residues as a precursor material. However, despite the growing number of experimental studies, predictive modeling of the thermal and structural evolution of the material during its conversion remains very limited. Physical activation takes place in two stages, carbonization and activation. Both processes are closely related and directly influence the final properties of the activated carbon. Therefore, it is essential to model both stages accurately.

This paper focuses on modeling the carbonization stage, which is considered critical in the overall activation process. The conditions under which carbonization occurs (temperature, heating rate, residence time, particle size) determine the char yield and its internal structure, elemental composition, and, ultimately, its ability to be efficiently activated in the second stage of the process. The final properties of activated carbon depend on the characteristics of the initial char. This highlights the need for kinetic models that reproduce the carbonization process.

Numerous approaches to modeling biomass pyrolysis can be found in the literature.^1,2^ These models can be classified as kinetic models and structural models. The formers describe the conversion of biomass to the main products (char, gas, and tar) using global or semiglobal schemes. The latter, more complex, are based on the molecular structure of the precursor and use bond networks and statistical functions to model thermal decomposition.^3–5^ Among the kinetic models, single-step global reaction schemes provide acceptable results and have been widely used for their simplicity. However, they have limitations because they do not adequately capture product distribution or structural changes throughout the process. Semiglobal mechanisms, on the other hand, define independent equations for the three main products (char, gas, and tar) to reflect the different proportions between them that arise depending on the decomposition temperature. Some only consider the generation of the three products but not the interaction between them.^6^ In contrast, others include the possibility that some byproducts can transform into others, such as tar into char and gases.^7,8^ Finally, some models aim to represent the different decomposition behaviors exhibited by the three pseudocomponents of biomass (cellulose, hemicellulose, and lignin). Numerous reaction schemes have been developed within this group for each pseudocomponent. For cellulose decomposition, stand out the classical proposals of Shafizadeh et al.,^9,10^ Piskorz et al.,^11^ and Banyasz et al.;^12^ for hemicellulose decomposition, the proposals of Shen et al. 2010,^13^ Patwardhan et al.,^14^ and Wang et al.;^15^ and for lignin decomposition, the proposals of Klein and Virk,^16^ Faravelli et al.,^17^ and Hou et al.^18^ Finally, the detailed schemes of Ranzi^19^ and Anca-Couce^20,21^ are highlighted, which consist of a multicomponent mechanism by which the superposition of different submechanisms describes biomass pyrolysis for each pseudocomponent of the biomass.

Faced with the limitations of single-step global reaction schemes and the complexity of detailed multicomponent semiglobal models, distributed activation energy models (DAEM) have become the most widely used approach in the scientific literature for describing biomass pyrolysis.^22^ The DAEM model assumes that thermal decomposition is not the result of a single reaction but rather a set of parallel and independent reactions (typically first-order), each characterized by a specific activation energy. These reactions can be represented by a continuous activation energy distribution function. Most recent works using the DAEM have focused in nonsymmetrical, fixed probability distributions, such as the Weibull, multiple Gaussian or logistic, and parameter optimization through advanced mathematical techniques.^23–29^ However, this involves imposing a specific functional form, which does not always agree with experimental data, especially in the case of biomass waste, which has a highly heterogeneous composition and, thus, affects the model’s accuracy.

Consequently, in this work, we develop a distribution-free DAEM model, in which the function f­(*E*) is directly derived from experimental data without assuming any specific distribution. This represents a relevant contribution of the study, since most DAEM-based approaches require the prior selection of a predefined activation energy distribution. The methodology proposed by Miura and Maki,^30,31^ which allows for determining both the activation energy distribution and the pre-exponential factor from TGA tests performed at different heating rates, was adopted to achieve this. This approach improves the model’s fitting capacity and allows for a more realistic characterization of the behavior of the conversion process. Furthermore, to solve the exponential integral present in the DAEM model equations, an alternative approximation for the integral temperature has been incorporated, which differs from those traditionally used in the literature (such as Doyle, Starink, or Murray-White). Based on the Abramowitz and Stegun expansion series, the new approximation used in the calculations presents a lower error, improving the model’s accuracy. This novel numerical treatment of the temperature integral is another specific contribution of the present work.

The kinetic characterization and validation of the model have been carried out through experimental tests with barley straw residues using thermogravimetric analysis under nonisothermal conditions at five different heating ramps. The use of five heating rates, instead of the customary three, also strengthens the kinetic identification and reduces uncertainty in the Arrhenius analysis. Barley straw was selected because barley is one of the main cereal crops in Europe, making this residue an abundant and relevant precursor for activated biocarbon production. However, detailed predictive modeling of its physical activation process remains limited. The results show a good fitting agreement between the experimental and simulated curves, validating the proposed model’s ability to describe the carbonization process under different operating conditions.

Finally, in future work, this model will be integrated into a comprehensive reaction model that includes the activation stage,^32^ allowing the full activated carbon production process to be simulated under industrial conditions. This comprehensive model will be a valuable tool for designing, optimizing, and scaling processes, both at the laboratory level and in industrial rotary drum reactors.

## 2 Materials and Methods

Barley straw, with one of the largest cultivated areas in Spain and Europe,^33^ was selected as the case study biomass. A considerable amount of this residual biomass (15–50%) has no end use, and it is incinerated in the field, which generates polluting emissions.^34^ The straw was collected during the harvest and mechanically baled, showing particles of various sizes and shapes, mostly from cane stems but also from leaves.

As a preliminary step for its characterization, the following pretreatments were carried out on a representative bale sample. First, it was dried in an oven at 105 °C until a moisture content of less than 10% was reached. Subsequently, the sample was ground using a hammer mill coupled to a vibrating sieve (size <0.5 mm). Finally, the standard quartering method (UNE-EN ISO 18135:2018) was used to classify into five size ranges (R1–R5) using standardized mesh screens (Table 1). Fraction R3 (0.250–0.150 mm) had the highest mass fraction (>35%), with an average size of 0.195 mm. The finest fraction (R5, <0.1 mm) accounted for 20% of the sample.

**Table 1:** Ultimate and Proximate Analysis in Barley Straw Samples R1-R5^a^

Size range/Average particle size (μm)			Ultimate (wt, %)^b^					Proximate (wt, %)^b^			
			C	H	N	O^c^	S	M	VM	FC^c^	Ash
R1	500–355	427	48.0	5.7	0.7	41.7	0.1	8.7	76.8	19.2	4.0
R2	355–250	302	47.7	5.7	0.8	40.7	0.1	8.8	75.9	19.1	5.0
R3	250–150	200	47.5	5.7	0.8	40.1	0.1	8.6	75.5	18.6	5.9
R4	150–100	125	47.1	5.7	0.9	40.2	0.0	8.5	75.3	18.6	6.1
R5	<100	50	46.5	5.7	1.3	37.7	0.0	8.4	74.3	16.9	8.8

a M – moisture; VM – Volatile matter; FC – Fixed carbon.

b Dry basis, except for moisture, which is on as received basis.

c Calculated by difference.

Regarding chemical composition, proximate and ultimate analyses in Table 1 were performed for each size fraction (EN/ISO standards). In addition, major and minor chemical elements present in the samples were determined using Inductively Coupled Plasma Optical Emission Spectrometry (ICP-OES). Proximate analysis shows decreased fixed carbon and volatile matter with decreasing particle size, while ash content increases from 4.0% (R1) to 8.8% (R5). Ultimate analysis shows that carbon and oxygen contents decrease slightly as particle size decreases, and sulfur content is almost zero. ICP-OES analysis revealed increased mineral content (K, Ca, Si, Mg, P) from R1 to R5, with Al and Fe being more abundant in the fine fractions, possibly due to external contamination during collection and handling. Further details of the effect of particle size distribution on physical properties and chemical composition can be found elsewhere.^35^

Finally, for the kinetic characterization of the thermal decomposition process, nonisothermal pyrolysis tests were carried out in a thermobalance (TG 209F1 Libra) under a nitrogen (N_2_) atmosphere, heating 7–9 mg of sample from room temperature to 900 °C at five heating rates (5, 10, 15, 20, 30 °C/min). The gas flow rate for all TGA tests was 50 mL/min.

### 2.1 Carbonization Model

A free-distribution DAEM model was chosen to define the carbonization stage. DAEM models are currently the most widely used approach, combining simplicity and accuracy for determining biomass pyrolysis. Compared to classical single-step global kinetic models or two-reaction competing models, DAEM models allow for a more accurate simulation of mass loss throughout the entire conversion process, providing a more realistic approximation by considering the reactions due to each type of chemical bond. The main hypothesis of the model is that thermal decomposition occurs through many independent and parallel reactions (typically first-order), with different activation energies that reflect variations in the bond strengths of the species involved. The number of reactions is so large that a continuous distribution function can describe the activation energies. A global reaction scheme represents particle conversion:

1 dα/dt=k(T)f(α)

where *t* is the time, *k­(T)* is the temperature-dependent reaction constant, and *f­(α)* is a function called the reaction model that describes the dependence of the reaction rate on the conversion, α. The conversion, α, is given by eq 2, where ω_*initial*_, ω_*final*_, and ω_*t*_ represent the sample weights at the beginning, at the end, and at time t during the reaction.

2 $$\alpha =\frac{\omega_{initial}-\omega_{t}}{\omega_{initial}-\omega_{final}}$$

The dependence of the conversion on temperature is introduced through the kinetic constant *k­(T)*, which presents an exponential Arrhenius-type behavior so that eq 1 can be written as

3 $$d\alpha /dt=A_{0}\exp\left(-E/RT\right)f\left(\alpha \right)$$

where *A*_0_ represents the pre-exponential factor, *E* is the apparent activation energy, and *R* is the universal constant of ideal gases (8.314 J/mol·K).

Assuming a constant heating rate β, characteristic of a nonisothermal heating process in TGA (*T* = *T*_0_ + *βt*), eq 3 can be rewritten as

4 $$d\alpha /dt=\left(A_{0}/\beta \right)\exp\left(-E/RT\right)f\left(\alpha \right)$$

Integrating these expressions leads to eq 5 and eq 6, respectively, where *g­(α)* is the integral form of the reaction model, and whose expressions constitute the basis for determining the kinetic parameters by integral methods.

5 $$g\left(\alpha \right)=\int_{0}^{\alpha }d\alpha /f\left(\alpha \right)=A_{0}\int_{0}^{t}\exp\left(-E/RT\right)dt$$

6 $$g\left(\alpha \right)=\int_{0}^{\alpha }d\alpha /f\left(\alpha \right)=\left(A_{0}/\beta \right)\int_{T_{0}}^{T}\exp\left(-E/RT\right)dT=\left(A_{0}/\beta \right)\left(\int_{0}^{T}\exp\left(-E/RT\right)dT-\int_{0}^{T_{0}}\exp\left(-E/RT\right)dT\right)$$

where $\int_{0}^{T}\exp\left(-E/RT\right)dT-\int_{0}^{T_{0}}\exp\left(-E/RT\right)dT\approx \int_{0}^{T}\exp\left(-E/RT\right)dT$ , since $\int_{0}^{T}\exp\left(-E/RT\right)dT\gg \int_{0}^{T_{0}}\exp\left(-E/RT\right)dT$ .

The integral ∫0Texp(−ERT)dT is called the temperature integral and has no analytical solution. Numerical methods or approximation solutions *p­(x)* can be used to solve it.^36–48^

$$g\left(\alpha \right)=\int_{0}^{\alpha }d\alpha /f\left(\alpha \right)=\left(A_{0}/\beta \right)\int_{0}^{T}\exp\left(-E/RT\right)dT=\frac{A_{0}E}{\beta R}p\left(x\right)$$ 7

where *x* represents *E*/*RT* and *p­(x)* is the approximate solution of the integral $\int_{0}^{T}\exp\left(-E/RT\right)dT$ .

8 $$g\left(\alpha \right)=\left(A_{0}/\beta \right)\int_{0}^{T}\exp\left(-E/RT\right)dT=-\frac{A_{0}E}{\beta R}\int_{x}^{\infty }\frac{\exp\left(-x\right)}{x^{2}}dx=\frac{A_{0}E}{\beta R}\underset{p\left(x\right)}{\underset{︸}{\left(\frac{\exp\left(-x\right)}{x}-\int_{x}^{\infty }\frac{\exp\left(-x\right)}{x}dx\right)}}$$

where $\int_{x}^{\infty }\frac{\exp\left(-x\right)}{x}dx$ is the exponential integral function *E*_*i*_ *(−x)*. Approximating the exponential integral function by the series expansion $E_{i}\left(x\right)\approx \frac{\exp\left(x\right)}{x}\left[1+\frac{1!}{x}+\frac{2!}{x^{2}}+\frac{3!}{x^{3}}+...\right]$ and retaining up to the second term, one of the most used approximations of the integral temperature *p­(x)* is obtained.^36^

9 $$p\left(x\right)≅\frac{\exp\left(-x\right)}{x}+E_{i}\left(-x\right)\approx \frac{\exp\left(-x\right)}{x^{2}}$$

Alternatively, the function *Ei­(x)*, for *x* > 0 can be represented by the Abramowitz and Stegun series (1964).^37^

$$\begin{array}{cc} E_{i}\left(x\right)= & -\gamma -\ln x-\overset{\infty }{\underset{n=1}{\sum }}\frac{{\left(-x\right)}^{n}}{n\;n!}\to \\ E_{i}\left(-x\right)= & \gamma +\ln x+\overset{\infty }{\underset{n=1}{\sum }}\frac{{\left(-x\right)}^{n}}{n\;n!} \end{array}$$ 10

where γ is Euler’s number. However, the convergence of the series is very slow for *x* > 2.5, and the result is imprecise. This series behaves as a negative exponential for large values of the argument and as a logarithm for small values, so that, for positive real values of the argument, *E*_*i*_ *(x)* can be bounded by elementary functions as follows:

11 $$\frac{1}{2}\exp\left(-x\right)\ln\left(1+\frac{2}{x}\right)<\underset{-E_{i}\left(-x\right)}{\underset{︸}{E_{i}\left(x\right)\;}}<\exp\left(-x\right)\ln\left(1+\frac{1}{x}\right)$$

So, the integral temperature $p\left(x\right)≅\frac{e^{-x}}{x}+E_{i}\left(-x\right)$ would also be bounded by elementary functions, which can be used for its approximation:

$$\begin{array}{c} \frac{\exp\left(-x\right)}{x}-\underset{lower}{\underset{︸}{\frac{1}{2}\exp\left(-x\right)\ln\left(1+\frac{2}{x}\right)}}>p\left(x\right)\\ p(x)>\frac{\exp\left(-x\right)}{x}-\underset{upper}{\underset{︸}{\exp\left(-x\right)\ln\left(1+\frac{1}{x}\right)}} \end{array}$$ 12

Many approximations of *p­(x)* have been proposed in the literature (Table 2). Among them, the first approach of Murray and White is the most widely used. In this work, however, the error obtained with the upper bound of the exponential integral function *E*_*i*_ *(−x)* was lower; therefore, this was the approximation adopted in the model.

**Table 2:** Approximations for Integral Temperature

Approach				Reference
$p\left(x\right)≅\frac{\exp\left(-x\right)}{x^{2}}$				Murray and White 1955 ^36^
$p\left(x\right)≅\exp\left(\frac{-Ax+B}{x^{k}}\right)$	A	B	k	
	1.0516	–5.3308	0	Doyle 1961 ^38^
	1	–0.235	1.95	Starink 2003 ^39,40^
	1.0008	–0.312	1.92	Starink 2003 ^39,40^
	1.000953	–0.297580	1.921503	Madhusudanan et al. 1993 ^41^
	1.000974	–0.299963	1.920620	Madhusudanan et al. 1993 ^41^
	1.001928	–0.389677	1.884318	Madhusudanan et al. 1993 ^41^
	1.00145033	–0.377773	1.894661	Tang et al. 2003 ^42^
$p\left(x\right)≅\left(\frac{\exp\left(-x\right)}{x}\right)\left(\frac{x^{3}+18x^{2}+88x+96}{x^{4}+20x^{3}+120x^{2}+240x+12}\right)$				Senun and Yang 1977 ^43^
$p\left(x\right)≅\frac{\exp\left(-x\right)}{x^{2}}\left(1-\frac{2}{x}\right)$				Coats and Redfern 1964 ^44^
$p\left(x\right)≅\frac{\exp\left(-x\right)}{x^{2}}\left(\frac{x}{1.0019882x+1.87391198}\right)$				Wanjun et al. 2003 ^45^
$p\left(x\right)≅\frac{\exp\left(-x\right)}{x^{2}}\left(\frac{x+0.6691}{x+2.64943}\right)$				Cai and Liu 2007 ^46^

Figure 1 shows the relative error of the temperature integral approximation adopted in this work compared with classical approaches reported in the literature, including Murray and White,^36^ Doyle,^38^ Starink (k = 1.92 and 1.95),^39,40^ and Coats and Redfern.^44^

**Figure 1:**
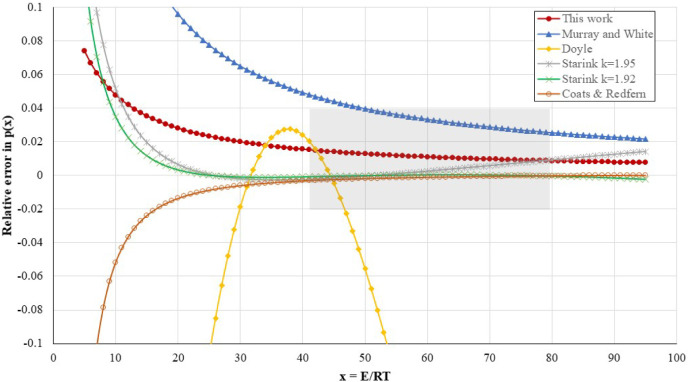
Relative error of different approximations for the temperature integral, p­(*x*). The shaded region indicates the range of interest for comparison in this study.

It can be observed that the approximation proposed in this work, based on the Abramowitz–Stegun expansion, yields consistently low and stable relative errors across the full range of *x*, and notably improves the accuracy with respect to the Murray and White approximation, commonly used in DAEM modeling studies. In contrast, Doyle’s approximation proves to be the least accurate, particularly at intermediate values of *x*, while the Starink (k = 1.95) and Coats and Redfern expressions provide improved overall accuracy.

Regarding the global reaction scheme of eq 1, the reaction model f­(α) describes how the reaction rate depends on the conversion. Numerous reaction models have been proposed for heterogeneous solid reactions (Table 3), and they generally fall into three main categories: increasing (power law), decreasing (reaction order), and sigmoidal.^49–51^

**Table 3:** Popular Reaction Models Used in Solid-State Kinetics

Reaction model	*f(α)*	*g(α)*
Power law 2	$2\alpha^{1/2}$	$\alpha^{1/2}$
Power law 3	$3\alpha^{2/3}$	$\alpha^{1/3}$
Power law 4	$4\alpha^{3/4}$	$\alpha^{1/4}$
Power law 2/3	$2/3\alpha^{-1/2}$	$\alpha^{3/2}$
Zero Order	1	α
First Order (Mampel)	1−α	−ln(1−α)
Second Order	${\left(1-\alpha \right)}^{2}$	${\left(1-\alpha \right)}^{-1}-1$
n^th^ Order	${\left(1-\alpha \right)}^{n}$	${\left(n-1\right)}^{-1}{\left(1-\alpha \right)}^{1-n}$
Sigmoidal (Avrami-Erofeev) 2	$2\left(1-\alpha \right){[-\ln\left(1-\alpha \right)]}^{1/2}$	${[-\ln\left(1-\alpha \right)]}^{1/2}$
Sigmoidal (Avrami-Erofeev) 3	$3\left(1-\alpha \right){[-\ln\left(1-\alpha \right)]}^{1/3}$	${[-\ln\left(1-\alpha \right)]}^{1/3}$
Sigmoidal (Avrami-Erofeev) 4	$4\left(1-\alpha \right){[-\ln\left(1-\alpha \right)]}^{1/4}$	${[-\ln\left(1-\alpha \right)]}^{1/4}$

Increasing models represent processes in which the reaction rate increases with conversion, reaching a maximum at the end of the process. This type of model can be described as a power law. Decreasing models represent processes in which the reaction rate peaks at the beginning of the process and the rate decreases as the conversion progresses. This model type can be described as a reaction order model, characteristic of biomass pyrolysis processes, where n is the reaction order. Finally, sigmoidal models represent processes in which conversion increases and decreases in the initial and final stages, reaching a maximum somewhere in between. These three types of behavior are easily distinguished under isothermal conditions since *k­(T)* = cte. However, under nonisothermal conditions, both *k­(T)* and *f­(α)* vary simultaneously, giving rise to sigmoidal curves (α vs *T*) in all cases.

Assuming a kinetics corresponding to the first-order reaction model (*f­(α)* = *(1 – α)*) and integrating for all the activation energies corresponding to each of the reactions that take place in the decomposition process following a distribution function *f­(E)*, the integral expressions of the DAEM model (eqs 13 and 14) that allows determining the total conversion of the particle are obtained:

13 $$\alpha =1-\int_{0}^{\infty }\exp\left[-\int_{0}^{t}A_{0}\exp\left(-E/RT\right)dt\right]f\left(E\right)dE$$

14 $$\alpha =1-\int_{0}^{\infty }\underset{\Phi \left(E,T\right)}{\underset{︸}{\exp\left[-\int_{0}^{T}\left(A_{0}/\beta \right)\exp\left(-E/RT\right)dT\right]}}f\left(E\right)dE$$

where *Φ­(E,T)* is the exponential integral function, representing the temperature integral of the Arrhenius expression.

Alternatively, differentiating to the equivalent differential expressions (eqs 15 and 16):

15 $$\frac{d\alpha }{dt}=\int_{0}^{\infty }A_{0}\exp\left[-\frac{E}{RT}-\int_{0}^{t}A_{0}\exp\left(-E/RT\right)dt\right]f\left(E\right)dE$$

$$\frac{d\alpha }{dt}=\int_{0}^{\infty }\left(\frac{A_{0}}{\beta }\right)\exp\left[-\frac{E}{RT}-\int_{T_{0}}^{T}\left(\frac{A_{0}}{\beta }\right)\exp\left(-E/RT\right)dT\right]f\left(E\right)dE$$ 16

In most of the literature, the distribution function is approximated by a Gaussian distribution function centered at *E*_0_ and with a standard deviation σ. However, it has the disadvantage of considering the distribution function to be symmetrical with respect to the mean when, in reality, it tends to be asymmetrical.^22^ To take this into account, some authors prefer to use other distribution functions, such as the Weibull or logistic distribution, or to construct a distribution function by linearly combining two or more Gaussian functions.^22,52,53^

Regarding the pre-exponential factor, three approximations are commonly referred to in the literature.^22^ The first is the conventional DAEM method, which assumes a constant value of the pre-exponential factor for all reactions. The second approximation expresses the dependence of the pre-exponential factor on temperature *A*_0_ = *A_c_T* ^*m*^, where *A*_*c*_ is a constant, and the exponent *m* varies between −1.5 and 2.5. Finally, the last approximation assumes that an increase in the activation energy is partially or wholly compensated by an increase in the pre-exponential factor (also called the “compensation effect” in the literature). In this case, the relationship between the activation energy and the most widely used pre-exponential factor is ln*A*_0_ = *aE* + *b*, where *E* is the mean value of the distribution function and *a* and *b* are constants.

### 2.2 Determination of Kinetic Parameters

Kinetic analysis of thermochemical processes aims to establish mathematical relationships between reaction rate, conversion, and temperature. To achieve this, it is necessary to experimentally determine the kinetic parameters of the reactions *A*_0_, *E*, and *f­(α)*, commonly referred to as the kinetic triplet.

The kinetic triplet is determined by fitting the reaction kinetics of the process to experimental data, generally obtained from TGA or DSC assays. To do this, the reaction equation is manipulated to obtain a characteristic expression that allows the kinetic parameters of the process to be determined by fitting the experimental data.^54^ A distinction is made between differential and integral methods depending on whether the reaction rate equation is used in its differential or integral form. Additionally, methods are classified as isoconversional or nonisoconversional based on whether they assume a specific kinetic model to evaluate the activation energy as a conversion function. In the literature, numerous methods can be found, the most popular being the Friedman method,^55^ Ozawa-Flynn-Wall method (OFW),^56,57^ Kissinger-Akahira-Sunose method (KAS),^58^ and Vyazovkin method^59^ within isoconversional models and the Coats-Redfern method^44^ within nonisoconversional models.

In this work, the integral methodology proposed by Miura and Maki^31^ was followed to determine the kinetic parameters (*f­(E)* and *A*_0_) of a free-distribution DAEM model. Compared to classical DAEM models that approximate the distribution function *f­(E)* by a known distribution function (typically Gaussian, Weibull, or Logistic), which does not always fit experimental data well, in this work, the distribution function *f­(E)* is obtained from experimental data, without assuming a specific distribution function. This method allows both *E* and *A*_0_ to be directly obtained from the Arrhenius plot. It does not require a differentiation procedure to obtain *dα/dT*, simplifying and making more precise the procedure for calculating *f­(E)* and *A*_0_ *(E)*.

The method of Miura and Maki assumes that, since the function *Φ­(E,T)* varies stepwise with activation energy at a given temperature, can be approximated by a step function at *E* = *E*_*s*_. Under this approximation, at a given temperature *T*, only a single first-order reaction with activation energy *E*_*s*_ is considered, where *E*_*s*_ satisfies the relation in eq 17. Consequently, the conversion expression is simplified according to eq 18.^31^

17 $$0.545\frac{\beta E_{s}}{A_{0}RT^{2}}=e^{-E_{s}/RT}$$

18 $$\alpha ≅1-\int_{E_{s}}^{\infty }f\left(E\right)dE=\int_{0}^{E_{s}}f\left(E\right)dE$$

By applying logarithms to the integral equation (eq 17), the characteristic equation of the method that allows determining the kinetic parameters of the process is obtained (eq 19).

19 $$\ln\frac{\beta }{T^{2}}=\ln\left(\frac{A_{0,\alpha }R}{E_{\alpha }}\right)+0.6075-\frac{E_{\alpha }}{RT}$$

To obtain the kinetic parameters in the integral method, experimental data for at least three heating rates, ln­(*β/T* ^2^) vs *1/T* at different degrees of conversion α, also called the Arrhenius plot, are plotted, and the fitting lines are calculated. The *E*_α_ values for a given conversion are obtained from the slope, and the corresponding *A*_0,*α*_ values are obtained from the intercept. Finally, using the *E*_α_ values obtained, α vs *E* are plotted, and the conversion is differentiated with respect to the activation energy to obtain the distribution function *f­(E)*.

## 3 Results and Discussion

### 3.1 TGA – Pyrolysis Tests

The Miura and Maki method establishes a minimum of three different heating ramps for determining kinetic parameters, the most common being between 3 and 5. Nevertheless, Soria-Verdugo et al.^60^ studied the influence of the number of TGA curves used in biomass pyrolysis to obtain kinetic parameters with the DAEM model. They concluded that if the uncertainties in the experimental measurements of heating rate and temperature are not considered in the Arrhenius plot’s linearization, at least five different heating ramps are required. This is necessary to obtain an average uncertainty of less than 10% when estimating the activation energy. For this reason, in this work, tests were carried out with five heating ramps (5, 10, 15, 20, and 30 °C/min) for each size fraction.

The thermal decomposition profiles obtained (Figure 2) show the typical behavior of lignocellulosic materials, with initial moisture loss (100 °C), followed by the sequential degradation of hemicellulose (300 °C), cellulose (350 °C), and lignin (500 °C). Although the general shape of the curves is similar across the different size fractions, it is observed that the finest particles (such as R5, <0.1 mm) exhibit shifts toward lower temperatures at the decomposition peaks, especially for cellulose. This effect is attributed to the greater presence of inorganic salts, such as potassium, which may catalyze pyrolysis by reducing the activation energy.^35^

**Figure 2:**
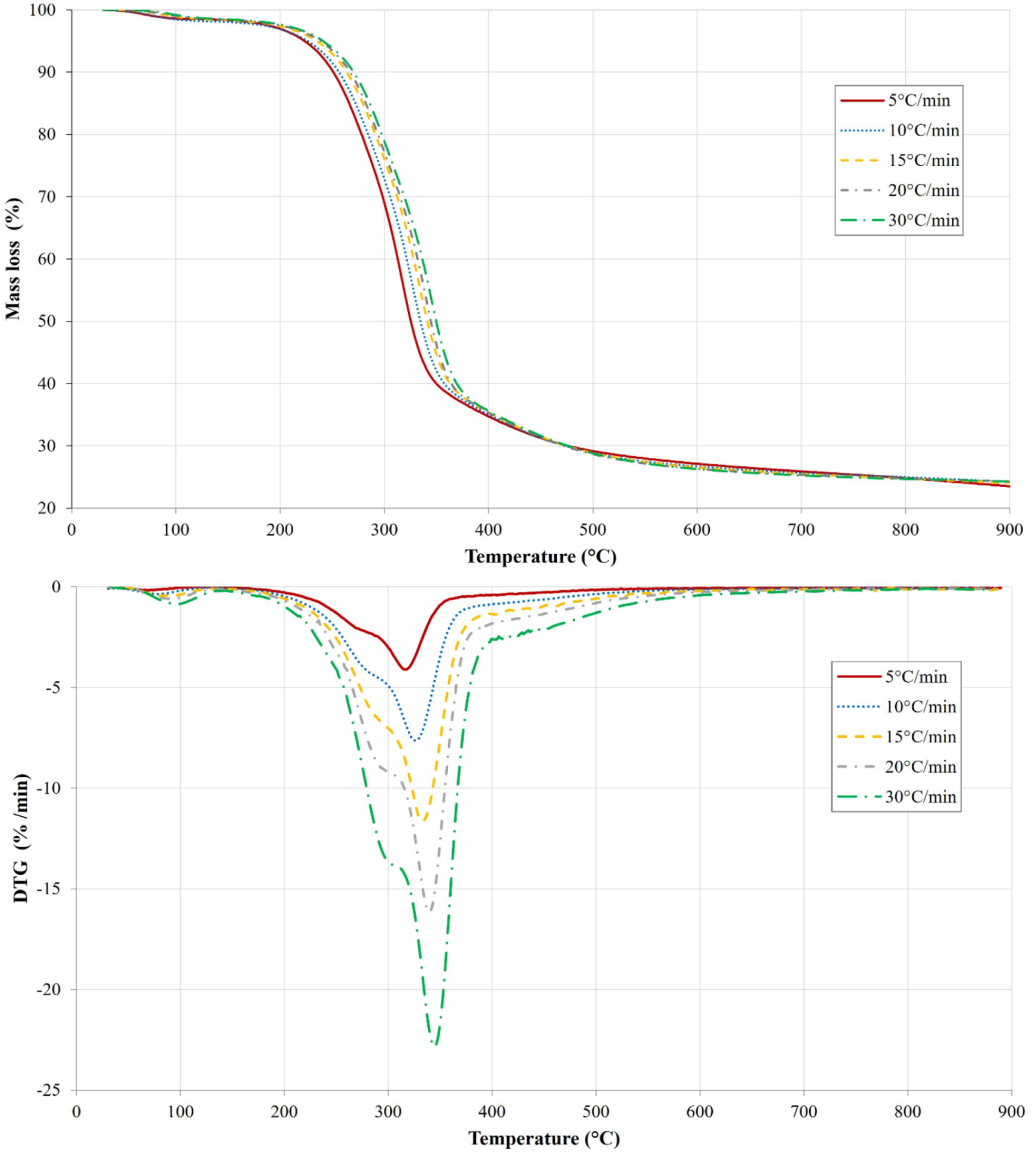
Mass loss (%) and DTG (%/min) evolution with temperature at five different heating rates (5–30 °C/min) for R3 fraction.

Furthermore, it is evident that as the heating rate increases, the thermal events shift toward higher temperatures, and the active pyrolysis zone widens, an effect associated with thermal inertia and heat transfer rates in the sample. In particular, lignin decomposition is enhanced above 400 °C. The differences observed in the final solid residue also reflect particle size: the finer fractions retain greater residual mass at 500 °C, correlating with their relative ash and fixed carbon content. Furthermore, at lower heating rates (5 °C/min), more complete lignin decomposition is favored in the range of 500 to 900 °C.

### 3.2 Kinetic Parameters

From the decomposition curves, the Arrhenius graph ln­(*β/T* ^2^) vs *1/T* has been represented at different degrees of conversion α (from 0.025 to 0.975 with a step of 0.025), obtaining the fitting lines for each conversion level; some of them are shown in Figure 3.

**Figure 3:**
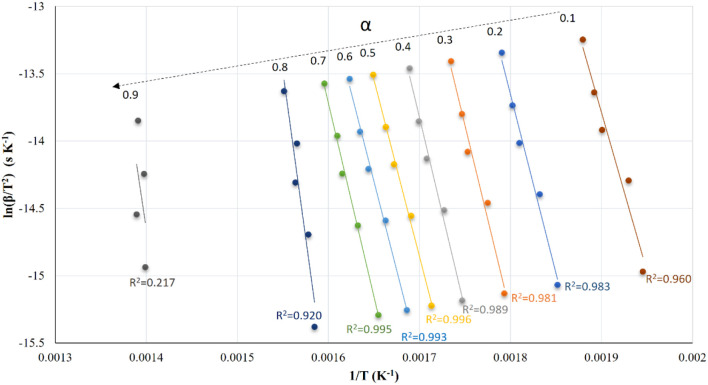
Arrhenius plot (ln­(β/T^2^) vs 1/T) for the R3 fraction at selected degrees of conversion (α = 0.1–0.9). The corresponding R^2^ values of the linear regressions are included for each conversion level.

The results show that, within the conversion range of 0.075 to 0.875, the Arrhenius plots exhibit satisfactory linearity, with R^2^ values above 0.9. However, lower correlation coefficients are obtained outside this range, indicating a poorer fit of the characteristic curves. This threshold was used as a practical criterion to identify the conversion range in which the isoconversional linearization is more consistent, in line with previous DAEM studies^61–64^ and other isoconversional models.^65,66^ On the one hand, during the initial stages of pyrolysis, the reactions are unstable, and regular activation energy values are not obtained.^64^ On the other hand, in the later stages of conversion, this behavior also indicates that multiple complex reactions occur simultaneously in the secondary pyrolysis of biomass.^62,63^ The DAEM model hypothesis, which assumes that only one first-order reaction occurs at each activation energy, would not be fulfilled out of this range.

The slope gives the *E*_α_ values for a given conversion, and the intersection with the *y*-axis gives the corresponding *A*_0,*α*_ values. Table 4 shows the *E*_α_ and *A*_0,*α*_ values obtained for each degree of conversion considered. The dependence of the activation energy on the conversion shows that pyrolysis occurs through multiple reactions.

**Table 4:** Activation Energies *E* and Pre-Exponential Factors A_0_ Obtained at Different Degrees of Conversion (α = 0.1–0.9) for the Five Size Fractions (R1–R5)

	R1		R2		R3		R4		R5	
α	*E* [kJ/mol]	*A*_0_ [s^–1^]	*E* [kJ/mol]	*A*_0_ [s^–1^]	*E* [kJ/mol]	*A*_*0*_ [s^–1^]	*E* [kJ/mol]	*A*_0_ [s^–1^]	*E* [kJ/mol]	*A*_0_ [s^–1^]
0.075	178.00	7.24 × 10^01^	228.97	3.98 × 10^02^	185.08	1.95 × 10^00^	230.80	2.16 × 10^–01^	186.46	1.72 × 10^04^
0.1	186.55	4.56 × 10^13^	239.96	1.56 × 10^20^	196.27	2.22 × 10^15^	217.68	1.58 × 10^21^	194.29	1.90 × 10^15^
0.125	190.20	8.98 × 10^15^	240.51	1.87 × 10^21^	201.89	8.30 × 10^16^	211.13	5.82 × 10^21^	190.99	2.30 × 10^17^
0.15	197.72	2.54 × 10^16^	234.10	7.50 × 10^21^	208.38	3.98 × 10^17^	212.05	7.15 × 10^19^	200.40	4.76 × 10^17^
0.175	207.15	2.96 × 10^16^	248.05	3.60 × 10^21^	213.15	6.69 × 10^17^	216.98	6.41 × 10^18^	198.62	9.27 × 10^16^
0.2	212.06	9.79 × 10^16^	248.22	4.15 × 10^20^	221.56	1.59 × 10^18^	228.57	4.13 × 10^18^	201.17	4.33 × 10^17^
0.225	210.08	5.17 × 10^17^	252.02	5.69 × 10^21^	225.52	2.75 × 10^18^	232.65	7.18 × 10^18^	206.00	1.62 × 10^17^
0.25	217.40	1.03 × 10^18^	252.30	3.55 × 10^21^	222.84	1.16 × 10^19^	232.80	5.94 × 10^19^	208.60	1.76 × 10^17^
0.275	227.29	4.48 × 10^17^	260.45	5.20 × 10^21^	235.36	1.81 × 10^19^	234.00	9.34 × 10^19^	211.72	3.37 × 10^17^
0.3	225.78	1.59 × 10^18^	269.91	3.57 × 10^21^	231.99	6.58 × 10^18^	242.78	6.22 × 10^19^	211.66	3.96 × 10^17^
0.325	231.91	9.56 × 10^18^	271.14	1.38 × 10^22^	240.40	6.97 × 10^19^	235.62	5.41 × 10^19^	216.14	5.31 × 10^17^
0.35	238.91	4.83 × 10^18^	259.81	7.07 × 10^22^	236.77	2.29 × 10^19^	240.37	2.44 × 10^20^	221.40	3.65 × 10^17^
0.375	238.97	1.27 × 10^19^	262.90	6.30 × 10^22^	237.92	9.70 × 10^19^	241.14	3.57 × 10^19^	220.76	6.95 × 10^17^
0.4	240.72	4.03 × 10^19^	259.06	3.74 × 10^21^	236.69	3.10 × 10^19^	226.51	6.92 × 10^19^	211.80	1.57 × 10^18^
0.425	238.15	2.96 × 10^19^	254.79	5.13 × 10^21^	230.81	2.86 × 10^19^	223.23	5.83 × 10^19^	219.13	1.01 × 10^18^
0.45	233.81	3.08 × 10^19^	245.63	1.62 × 10^21^	227.40	1.60 × 10^19^	218.19	1.89 × 10^18^	213.29	1.11 × 10^17^
0.475	231.59	1.35 × 10^19^	247.38	4.87 × 10^20^	222.03	3.44 × 10^18^	219.55	7.16 × 10^17^	209.50	4.13 × 10^17^
0.5	230.99	4.18 × 10^18^	238.38	5.41 × 10^19^	217.58	1.28 × 10^18^	215.12	1.92 × 10^17^	211.28	9.17 × 10^16^
0.525	221.54	2.05 × 10^18^	240.27	6.08 × 10^19^	217.73	3.28 × 10^17^	214.28	2.05 × 10^17^	207.49	3.36 × 10^16^
0.55	224.56	1.45 × 10^18^	239.96	7.32 × 10^18^	218.46	1.06 × 10^17^	207.96	6.54 × 10^16^	208.82	3.93 × 10^16^
0.575	226.44	1.67 × 10^17^	231.58	8.83 × 10^18^	220.16	8.92 × 10^16^	209.04	4.53 × 10^16^	208.15	1.46 × 10^16^
0.6	226.20	2.55 × 10^17^	233.33	6.81 × 10^18^	220.08	8.54 × 10^16^	208.98	1.02 × 10^16^	211.32	1.59 × 10^16^
0.625	228.09	3.11 × 10^17^	235.33	9.95 × 10^17^	225.28	1.01 × 10^17^	212.45	1.06 × 10^16^	214.10	1.15 × 10^16^
0.65	232.00	2.47 × 10^17^	239.30	1.17 × 10^18^	227.18	8.29 × 10^16^	215.90	8.83 × 10^15^	212.01	1.84 × 10^16^
0.675	237.33	3.01 × 10^17^	244.65	1.45 × 10^18^	226.92	1.96 × 10^17^	221.03	1.50 × 10^16^	219.11	2.69 × 10^16^
0.7	245.79	5.46 × 10^17^	254.37	2.66 × 10^18^	238.58	2.40 × 10^17^	231.97	2.52 × 10^16^	223.54	1.43 × 10^16^
0.725	244.29	1.30 × 10^18^	260.53	6.30 × 10^18^	250.65	1.82 × 10^17^	238.54	5.71 × 10^16^	234.20	4.86 × 10^16^
0.75	261.46	5.67 × 10^18^	287.04	3.47 × 10^19^	271.26	1.50 × 10^18^	255.21	4.18 × 10^17^	264.44	9.27 × 10^16^
0.775	270.40	3.27 × 10^18^	305.65	9.04 × 10^19^	308.69	1.27 × 10^19^	288.99	1.17 × 10^18^	308.87	5.79 × 10^17^
0.8	261.08	7.34 × 10^19^	297.81	1.24 × 10^22^	290.10	5.26 × 10^20^	273.24	2.26 × 10^19^	271.43	1.58 × 10^20^
0.825	314.20	2.99 × 10^20^	362.86	3.06 × 10^23^	308.05	4.87 × 10^23^	306.58	1.04 × 10^22^	280.51	5.01 × 10^23^
0.85	363.37	3.34 × 10^19^	380.02	4.07 × 10^22^	325.32	1.81 × 10^21^	345.69	2.66 × 10^20^	305.25	1.60 × 10^20^
0.875	443.50	4.62 × 10^23^	415.58	4.07 × 10^27^	389.41	4.82 × 10^22^	366.49	4.83 × 10^22^	312.08	2.72 × 10^20^

Figure 4 shows how activation energy values increase smoothly in the conversion range of 0.1 to 0.3, corresponding to the decomposition of hemicellulose and cellulose. Then, in the conversion range of 0.3 to 0.6, activation energy values remain stable or decrease smoothly. In this interval, the largest contribution corresponds to cellulose decomposition. Finally, they increase again in the final stage of conversion, where lignin conversion predominates in addition to the decomposition of residual cellulose, which presents greater stability and, therefore, higher activation energies.^64^

**Figure 4:**
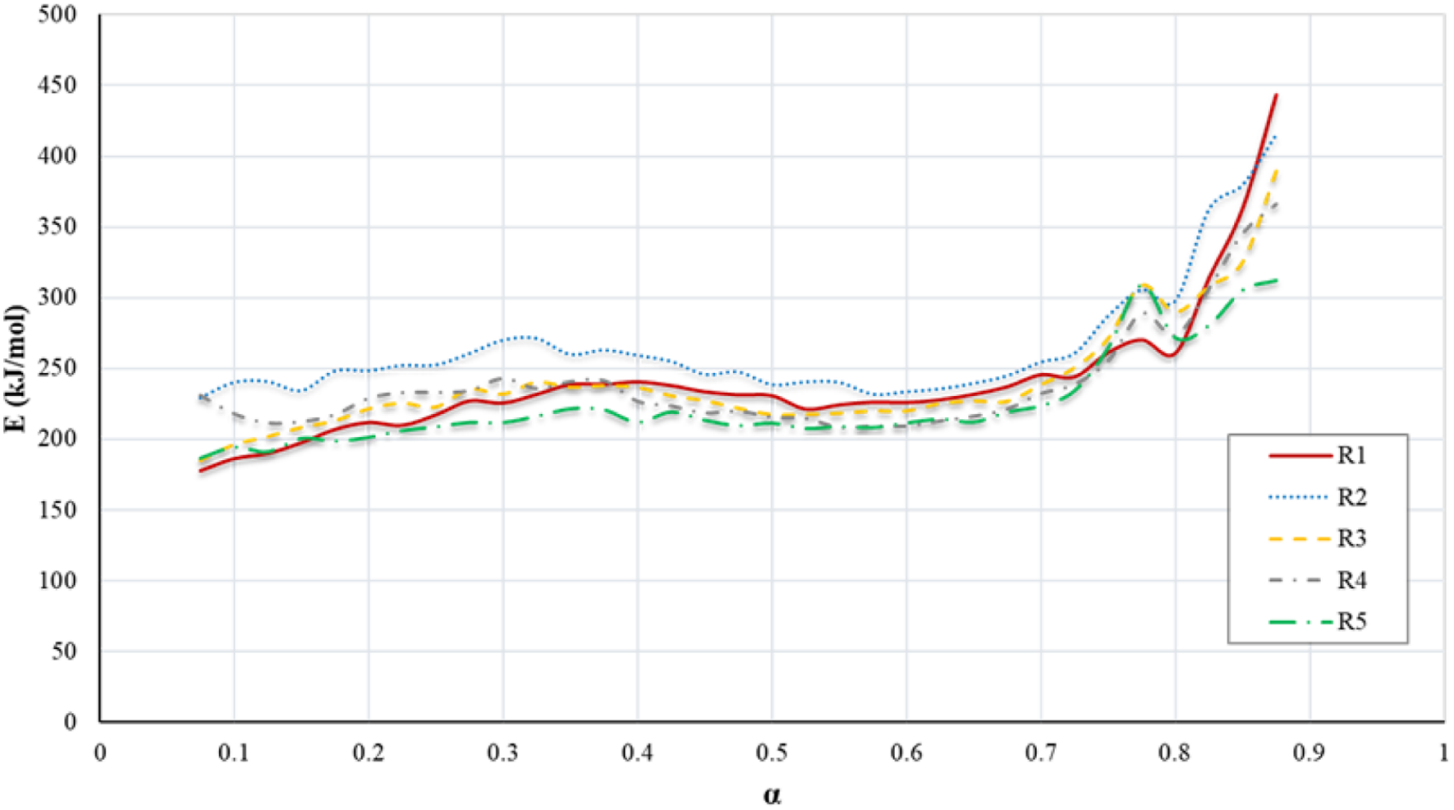
Activation energy (*E*) as a function of the degree of conversion (α) for the five particle size fractions (R1–R5).

This distribution is similar for all size fractions analyzed, but slightly shifted toward smaller activation energies as particle size decreases, presenting the following variation ranges: R1 (178–443 kJ/mol), R2 (229–415 kJ/mol), R3 (185–389 kJ/mol), R4 (230–366 kJ/mol) and R5 (186–312 kJ/mol). The greater reactivity of the smaller fractions observed in the experimental curves is therefore confirmed, due not only to the effects of the size of each fraction but also to the catalytic action of the alkaline elements (with greater presence in the smaller size fractions), causing a decrease in the temperature at which the cellulose peak occurs with particle size for all heating ranges. Other authors have pointed out this behavior,^61,67^ indicating that the catalytic effects of alkaline and alkaline earth metals during pyrolysis play an important role in the variation of the *f­(E)* curve.

The values obtained have been compared with other kinetic studies of pyrolysis with barley and similar lignocellulosic biomasses published in the scientific literature. Table 5 summarizes the models and fitting methods used in these publications and the results obtained for the activation energy. The dispersion of activation energy values can be attributed to several factors and, therefore, these values are not strictly comparable. First, differences in biomass composition, particularly in the relative contents of hemicellulose, cellulose and lignin, affect the conversion range in which each degradation process predominates.^70,71^ Second, the ash content and the presence of alkali and alkaline earth metals may catalyze pyrolysis reactions and shift the activation energy distribution toward lower values, as reported by Sonobe et al.^61^ and Várhegyi et al.^67^ Third, experimental conditions such as particle size, sample mass, heating rate and final temperature may influence heat and mass transfer effects and the contribution of secondary reactions. Finally, the kinetic method itself has a strong influence on the reported values, since model-free isoconversional methods,^51,66,73,74^ DAEM approaches based on predefined Gaussian distributions,^67,69–71^ and the Miura-Maki integral method^61,68^ do not provide directly equivalent kinetic parameters. Therefore, taking the differences in biomass species, mineral composition, experimental conditions and kinetic calculation methods into consideration, the activation energy ranges obtained in this work are in reasonable agreement with those reported for barley straw and other lignocellulosic biomasses. Focusing on the study most comparable to ours, Zhang et al. (2018),^68^ which also used barley straw, employed similar experimental TGA conditions and applied the same isoconversional fitting method, the results differ significantly from ours, both in the activation energy values and in how they vary with conversion.

**Table 5:** Summary of Kinetic Studies on the Pyrolysis of Lignocellulosic Biomasses

Reference	Material	Model/fitting method	Activation energy
This work	Barley straw	1st order DAEM	α = 0.075–0.875
		Miura/Maki integral method	*E*: 178–443 kJ/mol (R1)
			*E*: 229–415 kJ/mol (R2)
			*E*: 185–389 kJ/mol (R3)
			*E*: 230–366 kJ/mol (R4)
			*E*: 186–312 kJ/mol (R5)
Zhang et al. 2018 ^68^	Barley straw	1st order DAEM	α = 0.1–0.8
		Miura/Maki integral method	*E*: 73–214.11 kJ/mol
Sonobe et al. 2008 ^61^	Rice straw	1st order DAEM	
		Miura/Maki integral method	*E*: 118–208 kJ/mol
Várhegyi et al. 2009 ^67^	Barley, oats, wheat, and brassica straw	Two parallel reaction model 1st order DAEM (Gaussian)	
		Nonlinear fitting model	*E*: 167 and 231.5 kJ/mol
Várhegyi et al. 2011 ^69^	Wheat straw, sorghum, corn stalks, and rice husks	Two parallel reaction model 1st order DAEM (Gaussian)	
		Nonlinear fitting model	*E*: 176, 185, and 189 kJ/mol
Cai et al. 2013 ^70^	Wheat straw	Three parallel reaction model 1st order DAEM (Gaussian)	
		Nonlinear fitting model	*E*: 175.5, 204.2, and 240.61 kJ/mol
Chen et al. 2015 ^71^	Wheat straw	Three parallel reaction model 1st order DAEM (Gaussian)	*E*: 147.8, 167.98, and 195.18 kJ/mol
		Nonlinear fitting model	
Mani et al. 2010 ^72^	Wheat straw	Three parallel reaction model 1st order global kinetics	
		Nonlinear fitting model	*E*: 69, 78, and 80 kJ/mol
Cai and Bi 2009 ^73^	Wheat straw	1st-order global kinetics	α = 0.15–0.85
		FWO method	*E*: 133–173 kJ/mol
		Vyazovkin method	*E*: 130–171 kJ/mol
Mishra and Bhaskar 2014 ^51^	Wheat straw	-	α = 0.05–0.95
		Friedmann method	*E*: 146–345 kJ/mol
		KAS method	*E*: 139–282 kJ/mol
		FWO method	*E*: 141–280 kJ/mol
		Vyazovkin method	*E*: 139–290 kJ/kmol
Huang et al. 2016 ^66^	Sorghum straw	-	α = 0.1–0.7
		KAS method	*E*: 46–179 kJ/mol
		FWO method	*E*: 57–179 kJ/mol
Kongkaew et al. 2015 ^74^	Rice straw	-	α = 0.05–0.85
		KAS method	*E*: 177–220 kJ/mol
		FWO method	*E*: 177–221 kJ/mol

The differences in the activation energy values obtained may be due to the composition and particular characteristics of each residue. For instance, Zhang’s article does not include a complete characterization, including the inorganic element content of the samples. So those results must be considered with caution. To further explore this result, an additional comparison was made with the results of the kinetic study by Várhegyi et al. (2009),^67^ also conducted with barley straw. Based on the Gaussian distributions for the two reactions considered in their model, these are very similar to the free distribution obtained in our work. However, it should be noted that the activation energy values determined in their study were calculated for all the residues (not specifically for barley), and they followed a very different methodology. Since no other publications use barley straw, our results were compared with other pyrolysis studies using lignocellulosic biomasses. Table 5 shows that our results, for the same conversion range, are relatively similar, although again highlighting the dispersion in the results presented in the different publications, even for the same type of residue (see, for example, wheat straw).

Regarding the variation in activation energy with conversion, in Zhang’s study,^68^ the activation energy reaches a maximum for a conversion of nearly 0.6. In contrast, in this study, an increasing trend is observed in the later stages of decomposition. In the absence of a more detailed discussion in Zhang’s article, the evolution of the activation energy with conversion has been represented in Figure 5 in other works using isoconversional models with materials similar to barley straw. It can be observed that the evolution obtained in this work is similar to that obtained in most of these works (Cai and Bi 2009,^73^ Mishra et al. 2014,^51^ Kongkaew et al. 2015 ^74^), showing an increase in the last stages of the conversion. However, it is difficult to draw conclusions beyond this qualitative comparison due to differences in the composition of the waste, the specific characteristics of the tests performed, and the methodology followed to determine the kinetic parameters.

**Figure 5:**
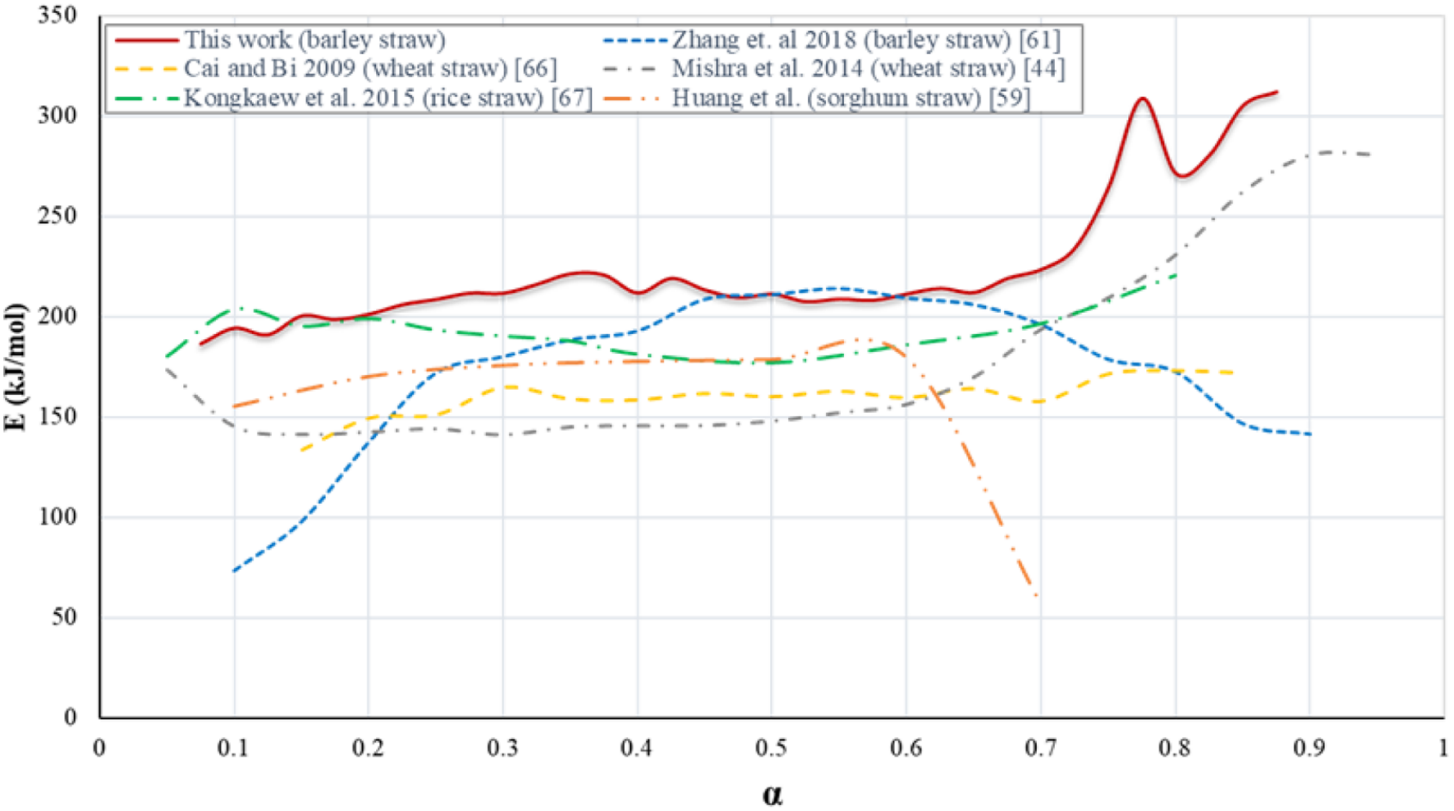
Comparison of activation energy (*E*) as a function of the degree of conversion (α) for the present work (barley straw) and literature data.

Once the isoconversional values of the kinetic parameters were obtained, differentiating the conversion α with respect to the activation energy *E* within the model fitting range (0.1–0.875), the activation energy distribution function *f­(E)* represented in Figure 6 was obtained.

**Figure 6:**
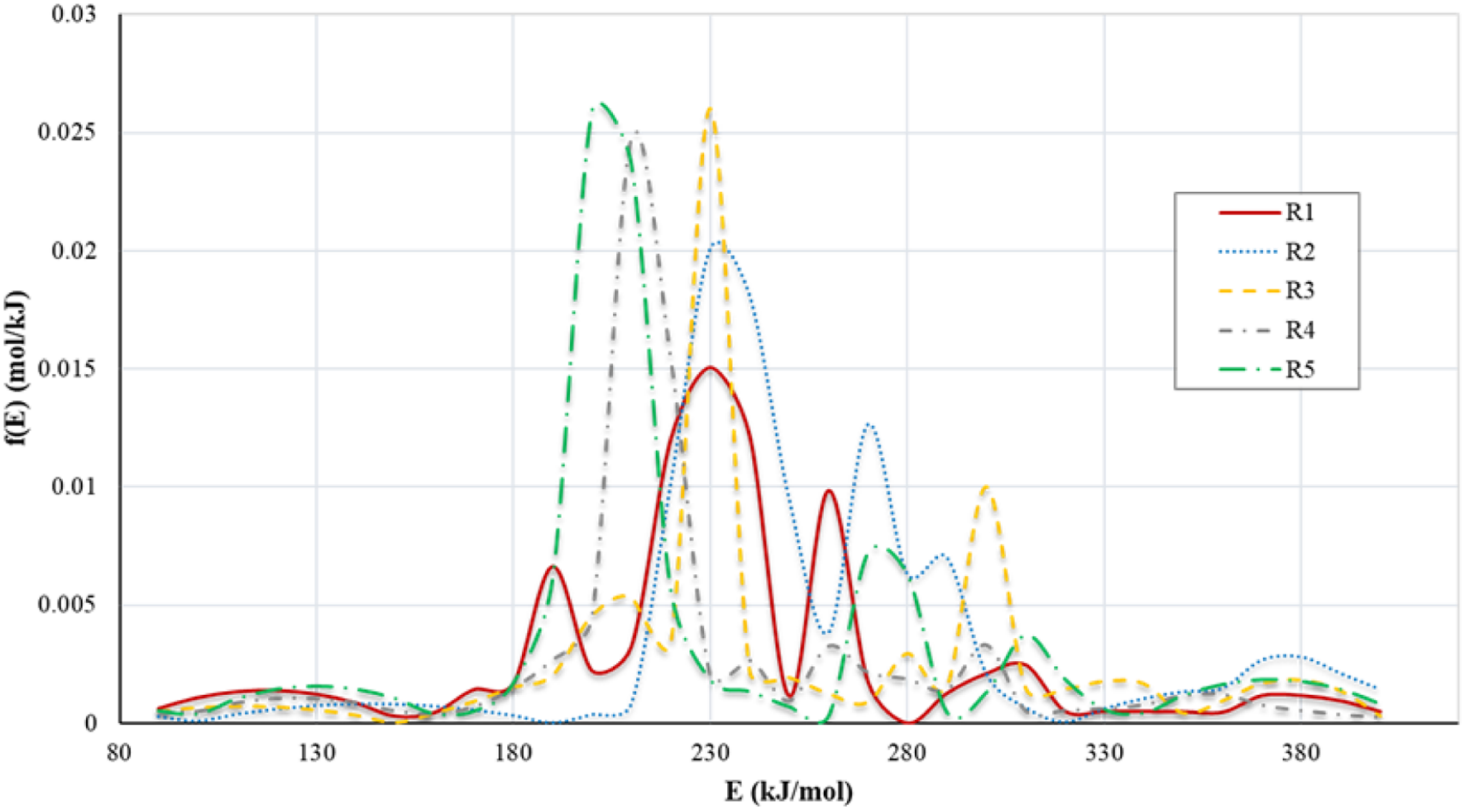
Activation energy distribution function, f­(*E*), for the five particle size fractions (R1–R5).

Although the kinetic parameters *E* and *A*_0_ vary with conversion, they exhibit a strong correlation called the compensation effect.^49^ Figure 7 shows the relationship between the pre-exponential factor and the activation energy. Within the considered conversion range, the relationship between ln *A*_0_ and E shows a reasonably linear trend, with R^2^ values above 0.9, suggesting the presence of a compensation effect (ln *A*_0_ = a*E* + b) in all cases, although slightly higher correlation coefficients are obtained for larger size fractions.

**Figure 7:**
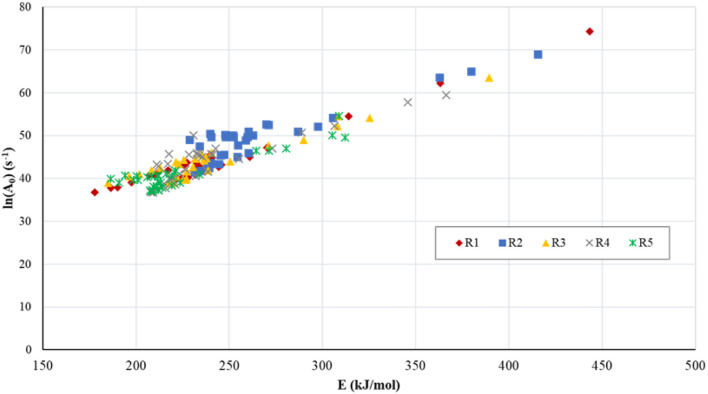
Relationship between ln­(*A*_0_) and activation energy (*E*) for the five particle size fractions (R1–R5).

Table 6 summarizes the values of the slope (a) and intercept (b), as well as the isokinetic temperature *T*_*iso*_ (a = 1/R*T*_*iso*_)^75^ and the corresponding rate constant *k*_*iso*_ (b = ln*k*_*iso*_)^75^ respectively, for all size fractions.

**Table 6:** Compensation Effect Parameters (a, b, *T_iso_, k_iso_*) for R1–R5 Size Fractions

	a	b	*T*_*iso*_ (K)	*k*_*iso*_ (s^–1^)
R1	0.14327	9.441	839.5	1.2590 × 10^04^
R2	0.14211	11.689	846.4	1.1920 × 10^05^
R3	0.15507	7.262	775.6	1.4249 × 10^03^
R4	0.13209	12.662	910.6	3.1550 × 10^05^
R5	0.11435	12.662	1051.9	5.1424 × 10^06^

From a thermodynamic point of view, the compensation effect has been related to the Gibbs free energy formulation and to the relationship between the entropy change and enthalpy change of the transition state,^76^ although this connection is difficult to demonstrate directly. However, an apparent compensation effect, also referred to as a false compensation effect, may arise from experimental uncertainties in the determination of rate constants as well as from the intrinsic mathematical correlation between kinetic parameters during regression.^76–78^

The interpretation of the origin of the compensation effect, whether physicochemical or artificial, remains unclear and is still a matter of debate.^50,75–78^ In this context, Agrawal^76^ pointed out that when the isokinetic temperature reaches unrealistically high values, the compensation behavior may simply be an artifact caused by non-negligible experimental and computational errors.

In the present work, the obtained isokinetic temperatures (775–1052 K) are slightly higher than the characteristic temperature range in which the main stage of pyrolysis occurs (500–700 K). This, together with the dispersion observed in both *T*_*iso*_ and *k*_*iso*_, suggests that the compensation effect is mainly associated with an apparent compensation effect due to experimental and computational uncertainties, rather than reflecting a true physicochemical compensation.

### 3.3 Prediction of Weight Loss Curves

Finally, using the kinetic parameters obtained for each size fraction R_i_, the conversion curves for different heating ramps were calculated with the proposed DAEM model and compared with the experimental TGA data, showing a good fit in all cases (Figure 8).

**Figure 8:**
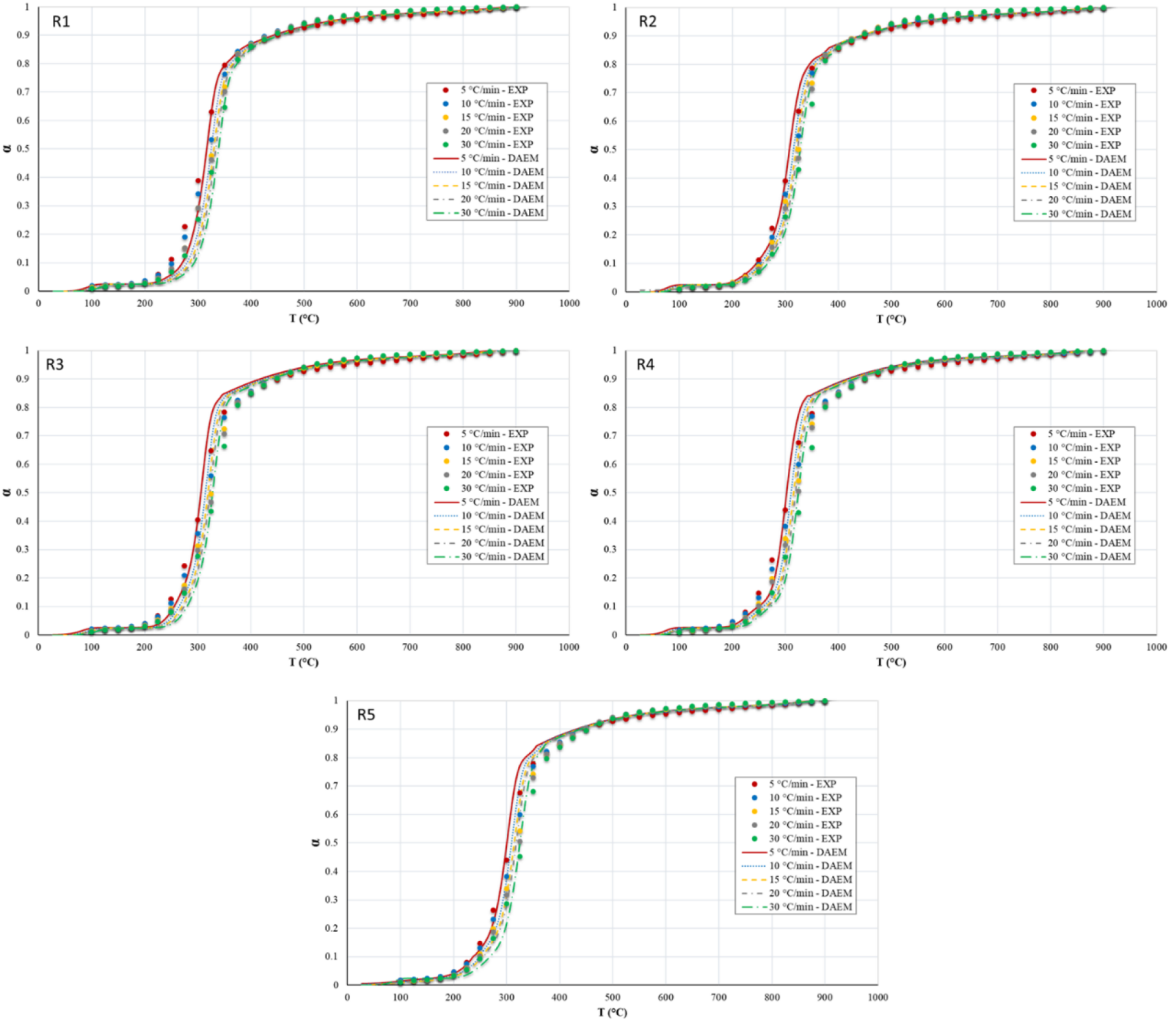
Comparison between experimental (markers) and calculated (lines) conversion (α) as a function of temperature for the five particle size fractions (R1–R5) at five different heating (5–30 °C/min).

Table 7 summarizes the statistical error indicators in terms of their range and mean values for each particle size fraction. The RMSE values ranged from 0.0147 to 0.0392, while the mean relative error (MRE) varied between 3.80% and 11.70%. On average, the MRE remained below 10% for all fractions, indicating a good agreement between experimental and model-predicted conversion curves.

**Table 7:** Summary of Statistical Error Indicators (Range and Mean Values) for Each Particle Size Fraction (R1–R5) across All Heating Rates (5–30 °C/min)

	MAE		RMSE		MRE (%)	
	(Range)	(Mean)	(Range)	(Mean)	(Range)	(Mean)
R1	0.0157–0.0217	0.0189	0.0328–0.0392	0.036	9.07–11.18	10.22
R2	0.0095–0.0122	0.011	0.0147–0.0184	0.0169	3.80–8.05	6.39
R3	0.0160–0.0212	0.0179	0.0288–0.0314	0.0298	8.41–9.60	8.87
R4	0.0157–0.0186	0.0173	0.0292–0.0313	0.0303	8.04–9.72	8.78
R5	0.0131–0.0169	0.0142	0.0207–0.0267	0.0228	4.38–11.70	6.71
		0.0159		0.0272		8.19

Among the analyzed fractions, R2 and R5 exhibited the lowest mean errors, whereas R1 showed slightly higher deviations. No clear trend with particle size was observed, suggesting that the model performance is not significantly affected by particle size variations. Overall, the relatively narrow error ranges and low mean values confirm the robustness of the proposed DAEM model.

Nevertheless, the model has intrinsic limitations. Regarding the accuracy of the Miura and Maki integral method followed in this work, Cai et al.^79^ conducted a study on this issue and concluded that due to the approximation of the *Φ­(E,T)* function as a step function, in some cases the method can produce significant errors in the estimation of the pre-exponential factor, and under certain conditions, the prediction of the distribution function may not be correct. In their discussion, they do not generally analyze under what conditions the prediction of *f­(E)* would be inaccurate, nor is there any subsequent work that delves into this aspect. Currently, the Miura and Maki integral method is the most widely used method for determining kinetic parameters in DAEM models. However, only values for the activation energy and the pre-exponential factor are obtained in a specific range of the conversion (typically 0.1–0.8).

To quantify the deviation associated with applying the DAEM assumptions to the initial and final stages of the process, additional simulations were carried out by extending the conversion range used for kinetic parameter estimation. Two additional scenarios were considered: inclusion of the initial conversion region and inclusion of the final conversion region. In each case, the kinetic parameters were recalculated and the DAEM simulations were repeated for the five heating rates.

The results confirmed that extending the analysis outside the range where satisfactory Arrhenius linearity was obtained increases the deviation between the experimental and simulated curves. For the selected conversion range, the overall statistical indicators were MAE = 0.0159, RMSE = 0.0272, and MRE = 8.19%. When the initial region was included, these values increased to MAE = 0.0455, RMSE = 0.0675, and MRE = 17.45%. When the final region was included, the increase was less pronounced, with MAE = 0.0337, RMSE = 0.0469, and MRE = 13.17%.

These results indicate that, in the present study, the deviation associated with extending the DAEM assumptions is more significant at the initial stages of the process. This behavior is consistent with the lower stability of the kinetic response at low conversion, where experimental deviations and preliminary overlapping phenomena may affect the Arrhenius linearity and the reliability of the estimated kinetic parameters.^64^ At the final stages, the observed deviation may be attributed to the contribution of secondary reactions occurring at high conversion.^62,63^

From an engineering perspective, the DAEM framework offers a suitable balance between model simplicity, computational efficiency, and predictive capability. In this context, the activation energy distribution f­(*E*) obtained in this work can be discretized, so that the double integral at each time step and for each discretized activation energy value can be computed using quadrature methods, enabling its implementation in reactor-scale or CFD simulations.^80–84^ Although the direct use of the continuous distribution may be computationally demanding, such simplified approximations provide a practical pathway for engineering applications. Therefore, despite its intrinsic limitations, the proposed approach constitutes a practical and efficient methodology, particularly suitable for processes such as biomass carbonization under moderate heating rates, where TGA conditions are representative of real systems.

## 4 Conclusions

This work presents a kinetic characterization of barley straw carbonization using a free-distribution DAEM approach combined with multirate thermogravimetric analysis, following the integral methodology proposed by Miura and Maki. The approach enables direct determination of the activation energy distribution f­(E) without assuming predefined functions. The results show that DAEM effectively captures the multistage behavior of biomass pyrolysis, with activation energies consistent with the literature.

The main contribution of this study lies in the rigorous implementation of a distribution-free DAEM with an improved numerical treatment of the temperature integral. The proposed approximation, based on the Abramowitz–Stegun expansion, yields consistently low and stable relative errors across the full range of conversion, compared to conventional formulations (e.g., Doyle or Murray and White). The use of five heating rates improves parameter robustness and reduces uncertainty in the Arrhenius analysis, in agreement with recent recommendations for DAEM-based kinetic identification. The model reproduces experimental conversion curves with acceptable accuracy across all particle size fractions, with mean relative errors within 6–10%.

Despite these strengths, the model presents inherent limitations. The assumption of independent first-order reactions may oversimplify the underlying mechanism, especially at low and high conversion levels where deviations are commonly observed. In addition, the continuous DAEM formulation is computationally demanding and typically requires simplification for reactor-scale applications.

From an engineering perspective, the DAEM approach offers a good balance between accuracy and simplicity, making it suitable for process modeling. However, its continuous formulation is difficult to apply directly in CFD or reactor-scale simulations. In practice, this limitation is usually addressed by simplifying the model, for example, by using discrete-DAEM or equivalent multireaction Arrhenius schemes. Within the context of this work, the model is well-suited for carbonization processes, where low heating rates make TGA results representative of real conditions. Overall, DAEM provides a reliable basis for kinetic modeling, but simplified versions are needed for industrial applications. More detailed models, such as multicomponent DAEM or structural approaches, can provide deeper insight but require more complex implementation and validation, often involving advanced characterization techniques and large data sets. This limits their use in engineering applications.

Future work will focus on integrating the carbonization model with a detailed activation-stage model and developing reduced kinetic representations for reactor and CFD simulations, enabling application to process design, optimization, and scale-up, particularly in rotary drum reactors.

## Supplementary Material



## Notes

The experimental thermogravimetric analysis (TGA) data for the five particle size fractions considered in this study, obtained under the five selected heating rates; the procedure used for the determination of the kinetic parameters by means of the Miura–Maki isoconversional method and the validation of the kinetic results using the distribution-free distributed activation energy model (DAEM) (XLSX)
